# Phase I/II study of melphalan, prednisone and lenalidomide combination for patients with newly diagnosed multiple myeloma who are not candidates for stem cell transplantation

**DOI:** 10.1038/bcj.2015.23

**Published:** 2015-03-20

**Authors:** V Roy, A K Stewart, P L Bergsagel, A Dispenzieri, K Laumann, J Allred, M Q Lacy, R Fonseca, C B Reeder, S Kumar, C E Rivera, M A Gertz, F K Buadi, S R Hayman, S V Rajkumar

**Affiliations:** 1 Hematology-Oncology, Mayo Clinic, Jacksonville, FL, USA; 2 Hematology, Mayo Clinic, Scottsdale, AZ, USA; 3 Hematology, Mayo Clinic, Rochester, MN, USA; 4 Biomedical Statistics and Informatics, Mayo Clinic, Rochester, MN, USA

Myeloma is a disease of older individuals, with median age at presentation of ~66 years.^1^ High-dose chemotherapy and autologous stem cell transplant is an important part of the treatment strategy but many patients are not candidates because of their inability to tolerate the rigors of high-dose chemotherapy.^2^ For many years, the cornerstone of multiple myeloma treatment in patients who are not candidates for autologous transplant was melphalan and prednisone combination (MP), which has only modest activity and low response rates, particularly complete remission rate.^3^ The availability of immunomodulatory drugs and proteasome inhibitors has led to remarkable improvement in outcomes of multiple myeloma, and several investigators have built on the MP backbone by adding these agents.^4, 5, 6, 7, 8^ Combination of melphalan, prednisone and thalidomide (MPT) was compared with MP in randomized trials and showed better response rates, improvement in progression-free survival and some trials also showed improved overall survival with MPT. However, MPT was associated with increased toxicity such as sedation, neuropathy and DVT.^9^ Thus, despite its efficacy, a concern about the toxicity and impairment of quality of life has hampered its widespread utilization in clinical practice. The combination of bortezomib, melphalan and prednisone was also associated with higher response rates, complete remission rate, progression-free survival and overall survival compared with MP in a randomized trial.^8^ However, risk of neuropathy and the inconvenience of parenteral administration of bortezomib are notable drawbacks of this regimen, particularly for an older patient.

Lenalidomide is a small molecule analog of thalidomide with potent immunomodulatory effects that is more active against myeloma compared with thalidomide, does not cause significant sedation and carries less risk of neuropathy or thromboembolism.^10, 11^ We hypothesized that that combination of MP and lenalidomide (MPR) will be an active anti-myeloma regimen with acceptable toxicity profile. We conducted a phase 1/2 trial to determine the maximum tolerated dose (MTD) and assess the tolerability and efficacy of the combination.

Patients over the age of 18 with previously untreated symptomatic myeloma by International Myeloma Working Group criteria^12^ and measurable or evaluable disease who were not candidates or declined autologous transplant were eligible. The study was approved by the Institutional Review Board of Mayo Clinic and all patients gave informed written consent prior to entry into the trial.

Study treatment consisted of lenalidomide (R) on days 1–21, melphalan (M) days 1–4 and prednisone (P) days 1–4. Cycles were repeated every 28 days till progression, intolerable side effects or withdrawal of consent. All patients received aspirin 325 mg a day for thromboprophylaxis. Routine antibacterial prophylaxis was not used. Stepwise reduction in doses of all the drugs was allowed per protocol for toxicity. The primary goal of the phase I part of the trial was to determine the MTD of this combination. Patients were accrued in cohorts of three at dose level 0 (M 5 mg/m^2^ day 1–4, P 60 mg/m^2^ day 1–4 and R 10 mg day 1–21) and dose level I (M 8 mg/m^2^, P 60 mg/m^2^ and R 10 mg/day). Patients were followed for at least two cycles to assess dose-limiting toxicity (DLT). G./GM-CSF was not allowed in the first two cycles unless DLT had occurred. The primary end point of the phase II part of the trial was to assess confirmed response rate using International Myeloma Working Group Uniform Response criteria.^13^ Toxicity of the regimen was evaluated as a secondary end point and graded according to Common Terminology Criteria for Adverse Events (CTCAE) v3.0.

Seven patients, median age 74 years (range 72–85), Eastern Cooperative Oncology Group (ECOG) status 0–1, were treated in phase I; four at dose level 0 and 3 at dose level I. All three patients at dose level I had DLT leading to the choice of dose level 0 as the recommended Phase 2 dose. Twenty-six additional patients (total 29) were treated at this dose. Their median age was 74 years (64–87), 17 (65%) were men. Seven (24%), 15 (52%), 6 (21%) and 1 (3%) patients had ECOG PS 0, 1, 2 and 3, respectively (Table 1). At the time of data freeze, the median follow-up of survivors was 36 months. A total of 209 treatment cycles were administered. Median number of cycles per patient was 8 (1–27). Neutropenia (48%/14%) and thrombocytopenia (17%/7%) were the most common grade 3/4 adverse events followed by anemia (17%/0%), fatigue (7%/4%), rash (14%/0%), and hyperglycemia (3%/0%). There were 31 treatment delays in 15 (52%) patients. Eleven, four and seven patients required dose reductions in L, P and M, respectively, most commonly because of neutropenia or thrombocytopenia. Eleven patients (42%) required G/GM-CSF treatment. All patients have completed study treatments; eight completed treatments per protocol, six refused further treatments, seven withdrew because of side effects, three withdrew because of unrelated illness, one chose alternative therapy and four for other reasons. Twenty (69% 95% CI 49–85) patients had a PR or better; four (14%) CR, seven (24%) VGPR and nine (31%) PR. Median time to response was 0.9 month. Median progression-free survival was 21.9 month (16.1–23.9) and overall survival at 1, 2 and 3 year was 90%, 66% and 59%, respectively.

We conclude that MPR combination in these doses (melphalan 5 mg/m^2^ day 1–4, prednisone 60 mg/m^2^ day 1–4 and lenalidomide 10 mg day 1–21; cycles repeated every 28 days) is feasible with a manageable toxicity profile and has significant activity in the treatment of patients with myeloma who are not candidates for stem cell transplant. Grade 3 or 4 neutropenia and thrombocytopenia was the most common toxicity, seen in up to 50% of patients in this cohort of predominantly elderly patients. No patient developed neuropathy or thromboembolic complications. Toxicity profile of lenalidomide compares favorably with those of thalidomide or bortezomib. Somnolence, sedation, constipation, thrombosis and neuropathy are predominant side effects of thalidomide, whereas neuropathy and the inconvenience of parenteral administration makes bortezomib unattractive, especially in elderly population. In contrast, lenalidomide is orally administered and associated with quite low risk of neuropathy or thromboses with appropriate thromboprophylaxis but appears to be more myelosuppressive. The optimal combination of MP and novel agents for any given patient may depend on pre-existing comorbidity. MPR regimen may be preferred for patients with pre-existing neuropathy, whereas patients with limited bone marrow reserve may not be suitable for this regimen. We recommend a well-designed randomized trial to carefully evaluate the efficacy and toxicity of these combinations including quality of life assessments.

## Figures and Tables

**Table 1:** Characteristics and outcomes of patients treated at phase II dose (*N*=29)

Age, median (range)	74 years (64–87)
Gender, male	17 (59%)
	
*ECOG performance status*	
0	7 (24%)
1	15(52%)
2	6 (21%)
3	1 (3%)
	
*ISS stage*	
I	5 (17%)
II	13 (45%)
III	11 (38%)
	
*Response rate*	
CR	4 (14%)
VGPR	7 (24%)
PR	9 (31%)

Abbreviations: CR, complete response; ECOG, Eastern Cooperative Oncology Group; ISS, international staging system; PR, partial response; VGPR, very good partial response.
